# Identification of a Cardiac Disease Modifier Gene Using Forward Genetics in the Mouse

**DOI:** 10.1371/journal.pgen.1000643

**Published:** 2009-09-18

**Authors:** William T. Pu

**Affiliations:** 1 Department of Cardiology, Children's Hospital Boston, Harvard University, Boston, Massachusetts, United States of America; 2 Harvard Stem Cell Institute, Harvard University, Cambridge, Massachusetts, United States of America; Harvard Medical School, United States of America

Heart failure, the clinical syndrome that arises from inadequate cardiac pump function, is known to be influenced by genetic factors. However, thus far unbiased genetic approaches in humans have met with limited success in identifying heart failure modifier genes [1], likely because of substantial genetic heterogeneity between patients and difficulty controlling environmental factors. These limitations can be overcome in model systems such as the mouse, where a wealth of inbred lines, polymorphic markers, and well-characterized heart failure models are available. Taking advantage of these resources, Wheeler and colleagues provide in this issue of *PLoS Genetics* compelling evidence that a little-studied, cardiac-specific protein kinase, *cardiac Troponin I-interacting kinase* (*Tnni3k*), is a strong modifier of the development of heart failure [2].

Previously, this team reported strain background–dependent differences in heart failure endpoints, including left ventricular contraction and survival, for the calsequestrin (*Casq1* or *CSQ*) transgenic model of dilated cardiomyopathy [3],[4]. Heart failure and death were accelerated when the CSQ transgene was crossed into the inbred C57BL6/J (B6) compared to the DBA/2J (DBA) strain background. The dominant B6 modifier locus, named *Hrtfm2*, was mapped to chromosome 3. Crosses between DBA/2J *CSQ* transgenic and AKR/J (AKR) mice identified a similar disease-accelerating locus mapping to the same chromosomal region, and haplotype analysis narrowed the candidate interval to 2 Mb, encompassing seven known genes [5]. In the present study, Wheeler et al. [2] demonstrate that cardiac expression of one of these genes, *Tnni3k*, is 12-fold higher in B6 and AKR than in DBA, and no significant differences were found in the other six genes. At the protein level, TNNI3K is robustly expressed in B6 and AKR hearts, but not detected in DBA hearts. This difference in expression is likely due to an intronic SNP in DBA that causes aberrant RNA splicing and production of a frameshifted transcript, which in turn is degraded by nonsense-mediated decay. Two lines of evidence indicate that *Tnni3k* is the gene underlying the *Hrtfm2* locus. First, while transgenic expression of *Tnni3k* in a largely DBA background did not affect heart function or survival in control mice, it markedly accelerated heart failure in *CSQ* transgenic mice. Second, congenic mice containing the AKR *Hrtfm2* region isolated in a DBA background exhibited both increased *Tnni3k* expression and accelerated death of *CSQ* transgenics.

Discovery of *Tnni3k* as a modifier of murine heart failure identifies it as a candidate for development of drugs to treat heart failure. Its promise as a pharmaceutical target is enhanced by virtue of its cardiac-restricted expression and by the potential “drugability” of kinases. However, pursuit of *Tnni3k* as a therapeutic target will require better understanding of its endogenous function and additional data linking of *Tnni3k* to heart disease in humans. *Tnni3k* encodes a cardiac-restricted kinase that belongs to the MAPKKK family, with homology to integrin-linked kinase (*Ilk*), *Raf1*, and *Tak1* (MGI: *Map3k7*) [6]. The similar domain structure of TNNI3K and ILK and the interaction of TNNI3K with cardiac troponin I (TNNI3) has suggested that TNNI3K connects integrin signaling with the sarcomere [6]. TNNI3K localized to both the cytoplasm and the nucleus [6], raising the possibility that TNNI3K engages in nuclear-cytoplasmic shuttling, coupling signals transduced in the sarcomere with transcriptional responses. Interestingly, DBA mice that do not express detectable TNNI3K protein are overtly normal. Presumably, TNNI3K structure and cardiac-restricted expression pattern have been preserved through evolution because they provide a selective advantage. As with the renin-angiotensin and β-adrenergic systems, inhibition of which are mainstays of current heart failure therapy, *Tnni3k* may participate in adaptive stress responses that become deleterious in pathological conditions such as heart disease.

While the work of Wheeler et al. [2] clearly links *Tnni3k* with accelerated disease progression in the *CSQ* heart failure model, a crucial question is whether this finding can be extrapolated to human disease. Significant additional work will be required to formally demonstrate that *Tnni3k* activity modulates progression of human heart disease, but two sets of data suggest that the results may be relevant. First, *CSQ* mice model many aspects of human dilated cardiomyopathy. Overexpression of *CSQ*, the major calcium-binding protein of the sarcoplasmic reticulum, disrupts cardiomyocyte calcium handling by reducing Ca*^2+^*-induced Ca*^2+^* release and altering expression of Ca*^2+^*-uptake and -release proteins [7]. Calcium is a central regulator of cardiomyocyte growth and function, and CSQ mice develop some calcium-handling abnormalities that are a common feature of diseased cardiomyocytes [8]. Second, Wheeler et al. [2] examined the effect of elevated *Tnni3k* expression in an independent disease model, in which heart failure was induced by constricting the transverse aorta. *Tnni3k* exacerbated cardiac dysfunction in this pressure-overload model, thereby providing important evidence that *Tnni3k* regulates development of heart disease in a model that may be physiologically more directly relevant to human heart disease.

As exemplified by the study of Wheeler et al. [2], exploration of genetic variation in mice can yield novel insights into human disease mechanisms. At present this resource has been underutilized, despite the fact that it directly complements analysis of genetic variation in human populations. Hopefully, improved mapping tools, increasing availability of strain-specific phenotyping data (e.g., http://www.jax.org/phenome [9]), and successful studies such as that of Wheeler et al. [2] will lead to more widespread use of forward genetic approaches in mice to inform translational research.
